# CRISPR/Cas9-Mediated Correction of the *FANCD1* Gene in Primary Patient Cells

**DOI:** 10.3390/ijms18061269

**Published:** 2017-06-14

**Authors:** Karolina Skvarova Kramarzova, Mark J. Osborn, Beau R. Webber, Anthony P. DeFeo, Amber N. McElroy, Chong Jai Kim, Jakub Tolar

**Affiliations:** 1 Department of Pediatrics, Division of Blood and Marrow Transplantation, University of Minnesota, Minneapolis, MN 55455, USA; karolina.skvarova@gmail.com (K.S.K.); webb0178@umn.edu (B.R.W.); apdefeo@umn.edu (A.P.D.); leira001@umn.edu (A.N.M.); tolar003@umn.edu (J.T.); 2 Childhood Leukemia Investigation Prague (CLIP), Department of Pediatric Hematology and Oncology, Second Faculty of Medicine, Charles University, Prague 15006, Czech Republic; 3 Stem Cell Institute, University of Minnesota, Minneapolis, MN 55455, USA; 4 Center for Genome Engineering, University of Minnesota, Minneapolis, MN 55455, USA; 5 Asan-Minnesota Institute for Innovating Transplantation, University of Minnesota, Minneapolis, MN 55455, USA; 6 Asan Institute for Life Sciences, Asan Medical Center, Asan-Minnesota Institute for Innovating Transplantation, Seoul 138-736, Korea; ckim@amc.seoul.kr

**Keywords:** gene editing, CRISPR/Cas9, Fanconi anemia, fibroblasts, Fanconi anemia D1, poly adenosine diphosphate-ribose polymerase inhibitors

## Abstract

Fanconi anemia (FA) is an inherited condition characterized by impaired DNA repair, physical anomalies, bone marrow failure, and increased incidence of malignancy. Gene editing holds great potential to precisely correct the underlying genetic cause such that gene expression remains under the endogenous control mechanisms. This has been accomplished to date only in transformed cells or their reprogrammed induced pluripotent stem cell counterparts; however, it has not yet been reported in primary patient cells. Here we show the ability to correct a mutation in Fanconi anemia D1 (*FANCD1*) primary patient fibroblasts. The clustered regularly interspaced short palindromic repeats (CRISPR)/Cas9 system was employed to target and correct a *FANCD1* gene deletion. Homologous recombination using an oligonucleotide donor was achieved and a pure population of modified cells was obtained by using inhibitors of poly adenosine diphosphate-ribose polymerase (poly ADP-ribose polymerase). *FANCD1* function was restored and we did not observe any promiscuous cutting of the CRISPR/Cas9 at off target sites. This consideration is crucial in the context of the pre-malignant FA phenotype. Altogether we show the ability to correct a patient mutation in primary *FANCD1* cells in a precise manner. These proof of principle studies support expanded application of gene editing for FA.

## 1. Introduction

Fanconi anemia (FA) is an inherited genetic disorder characterized by chromosomal instability, bone marrow failure (BMF), congenital malformations, and early cancer onset [1,2,3]. FA is caused by perturbations to one of the ~21 described genes that participate in interstrand cross link DNA lesion repair [2]. As a monogenic disorder FA represents an ideal candidate for phenotypic rescue by gene therapy or gene editing. A key consideration, in the pre-malignant FA phenotype, is safety of the intervention. Random or semi-random integration of gene therapy vehicles [4,5,6,7,8] with unregulated gene expression may be contraindicated in FA. The ability to modify genomic sequences in a precise and targeted manner represents a powerful approach for individualized translational medicine. Programmable nucleases (zinc-finger nucleases (ZFN) [9], transcription activator-like effector nucleases (TALENs) [10,11] or clustered regularly interspaced short palindromic repeats (CRISPR)/Cas9 [12,13]) have proven to be extremely useful for disrupting clinically relevant genes or correcting disease-causing mutations. Previous efforts for precision genome engineering in FA have been described for the Fanconi anemia A, C, and I gene mutations, respectively [14,15,16]. Common to each of these studies was the requirement of transformative factors such as telomerase reverse transcriptase expression [14] or pluripotency reprogramming genes [16]. To determine whether the FA phenotype could be corrected in true primary cells we undertook efforts to target the *FANCD1* gene in fibroblasts derived from an FA patient. *FANCD1*, also known as the breast cancer 2 gene (*BRCA2*), functions as a downstream effector of the DNA repair pathway where it binds and stabilizes RAD51-nucleoprotein filaments at the site(s) of DNA breaks [17]. This is a crucial prerequisite for DNA damage repair by homologous recombination [18,19]. Compromisation of this pathway results in a severe form of FA with significantly earlier onset of the disease manifestations and heightened incidence of brain tumors, nephroblastoma, and leukemia [20,21]. The disease phenotype, with extension to other tumors with impaired *BRCA* activity, shows a sensitivity to poly(ADP-ribose) polymerase (PARP) inhibitors [22]. We hypothesized that genome editing along with PARP inhibition (PARPi) would allow us to selectively recover gene modified cells. To accomplish this we delivered CRISPR/Cas9 reagents and an oligonucleotide donor molecule to primary *FANCD1* fibroblasts and, following PARPi treatment, obtained six genotypically and functionally corrected clones. To our knowledge, this is the first report of a causal gene correction by gene editing in FA patient primary fibroblasts.

## 2. Results

### 2.1. Design and Activity of CRISPR/Cas9 Gene-Editing Reagents for FANCD1 Gene Correction

**Figure 1:**
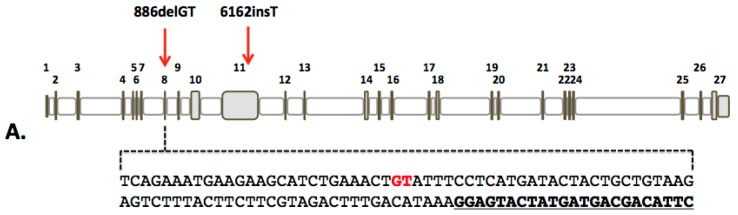

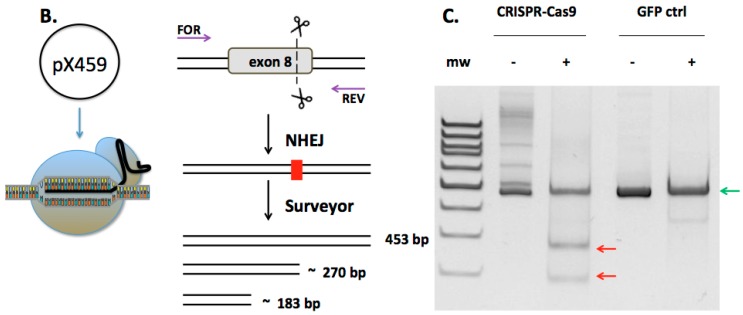
*FANCD1* gene and CRISPR/Cas9 reagent design. (**A**) *FANCD1* genomic architecture with causative mutations shown with red arrows. The full exon eight sequence is shown with the bases deleted in the 886delGT mutation highlighted in red. The CRISPR/Cas9 target sequence is shown in bold and underlined; (**B**) CRISPR/Cas9 construct and Surveyor assay. The pX459 DNA plasmid contains both the Cas9 and the gRNA that when delivered are expressed and form the functional complex. Following plasmid delivery, a region of the *FANCD1* locus was amplified using the primers indicated by purple arrows (forward (FOR) and reverse (REV)). CRISPR/Cas9 activity causes gene repair by non-homologous endjoining (NHEJ; red box). The Surveyor enzyme cleaves hybridized heteroduplexes of modified and unmodified amplicons; (**C**) 293 cell Surveyor analysis. The cleavage products showing *FANCD1* gene modification are shown with red arrows. GFP treated controls are at right. The green arrow shows the full-length amplicon. “+” = addition of Surveyor enzyme; “−” = no Surveyor enzyme; “mw” = molecular weight standards.

We derived primary fibroblasts from a minimally invasive punch biopsy from the extremity of a pediatric patient who has compound heterozygous mutations (886delGT and 6162insT) in the *FANCD1* gene. Because carrier patients are unaffected, heterozygous modification can be employed for phenotypic rescue. The 886delGT was favorable for designing reagents derived from the Streptococcus pyogenes CRISPR/Cas9 system due to the proximity of the guide RNA (gRNA) to the mutation site in exon 8 (Figure 1A). We assembled the exon 8 targeting gRNA and delivered it *in cis* on a DNA plasmid with Cas9 and tested it using the Surveyor method [23] (Figure 1B). Fragmentation products consistent with genome modification were observed following polyacrylamide gel electrophoresis (Figure 1C).

### 2.2. FANCD1 Gene Correction in Primary Patient Fibroblasts

**Figure 2:**
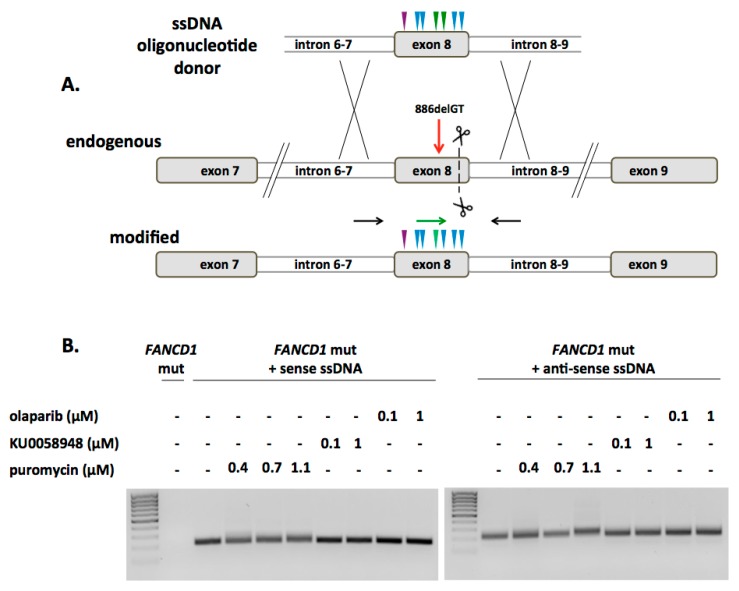
*FANCD1* gene correction. (**A**) Gene correction experimental design. A single stranded oligonucleotide donor spanning exon 8 and adjacent intron sequence was designed to have polymorphic sequences. These polymorphisms prevent re-cutting of the donor (blue arrows), add the GT dinucleotide (green arrow), and insert a *Hind* III restriction site (purple arrow); (**B**) HDR screening. Cells were unselected or selected in bulk with 1 μM olaparib, 1 μM KU0058948, or 1.1 μM puromycin. PCR with primers that are within the donor (green arrow) and outside the donor in exon 8 (black arrow in (**A**) were used to amplify the modified locus.

In order to facilitate gene repair, we considered both double and single stranded repair templates. The advantage of double stranded DNA (dsDNA) donors is their stability; however they have an associated risk of random insertion. Single stranded oligonucoleotide donors (ODN) can be delivered at a higher molar concentration; however, their linear configuration may make them more labile. To determine the rates of HDR mediated by each template we employed the traffic light reporter assay [24] and observed 1.5% and 4.5% HDR rates for ODN and dsDNA donors, respectively (Figure S1). Given the genomic instability associated with FA and the concern for ectopic integration of dsDNA we focused on ssODN donor optimization for gene correction. First, we tested whether a strand preference was evident when using positive or negative strand ODN donors. By using *FANCD1* locus flanking sequences encompassing a unique sequence that allowed for PCR primer design we observed higher rates of ODN donor incorporation when using a plus strand donor in 293T cells (Figure S2). To determine if a strand bias was operative in FA we targeted the 886delGT mutation with either sense or anti-sense donors (Figure 2A). The ODNs possessed approximately 60 bp of homology to the *FANCD1* locus on either side of the CRISPR/Cas9 protospacer adjacent motif. The donor’s also included six silent single-nucleotide polymorphisms (SNPs), one of them creating a *Hind*III restriction enzyme site (Figure S3). To increase the stability of the corrective ODNs, two phosphorothioate bonds were added at the 5′ and 3′ termini [25]. After sense or anti-sense ODN and Cas9: gRNA plasmid delivery we applied selective pressure to the bulk population of cells using puromycin, olaparib, or KU0058948 an olaparib analog (Figure 2B). Because the pX459 Cas9 plasmid contains a puromycin resistance gene we employed puromycin to enrich for cells that received the targeting molecules. The PARP inhibitor olaparib or KU0058948 were added in order to select for phenotypically rescued cells. These bulk populations of cells were analyzed for ODN mediated HDR using a primer specific for the donor SNPs. Each of the treatment groups showed donor incorporation using this sensitive assay (Figure 2B).

**Figure 3:**
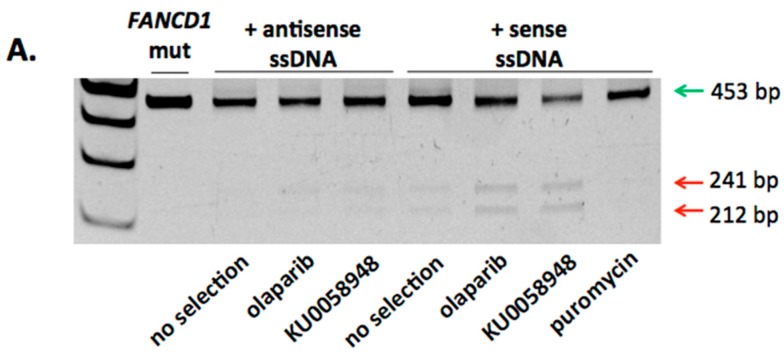

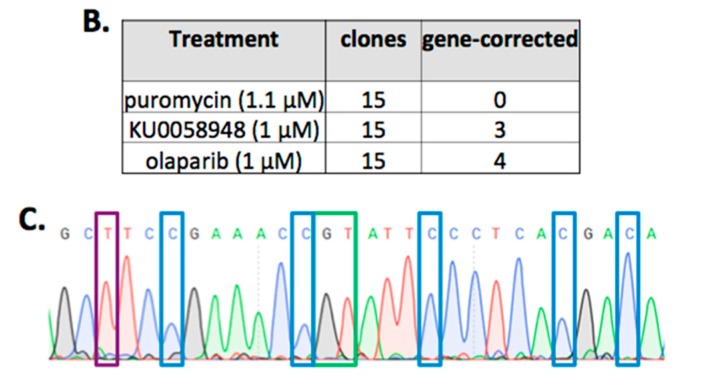
FANCD1 selection and clonal derivation. (**A**) Screening of sense and anti-sense ODN treated cells. PCR with primers that bracket exon 8 (shown as black arrows in Figure 2A were used to amplify the locus (green arrow, 453 bp) followed by *Hind*III digest. HDR results in a *Hind*III site that results in ~200 bp fragments (red arrows); (**B**) Clonal isolation. Limiting dilution cloning was performed and the individual clones were screened for HDR. Selection treatment is shown at left, middle panel indicates the number of clones, and right panel shows the frequency of HDR observed in the clones. (**C**) Corrected *FANCD1* locus. A representative Sanger sequencing chromatogram is shown for clones that underwent HDR. The blue boxes are the introduced silent polymorphisms that make the modified locus resistant to nuclease re-targeting. The purple box shows the *Hind*III site, and the GT dinucleotide that corrects the gene is within the green box.

Following this, we utilized locus specific primers that did not bind donor sequence and subjected the amplicons to *Hind*III digest. Using this approach, we observed that the sense strand donor showed the highest modification rates (Figure 3A). Based on these initial screening results, subsequent limiting dilution clonal derivation was undertaken. Forty-five total clones were obtained (15 clones per cell population of puromycin, KU0058948, or olaparib selection) and analyzed by PCR. HDR with correction of the 886delGT sequence was observed in 3/15 clones selected by KU0058948 and 4/15 clones treated with olaparib (Figure 3B,C). These data show the ability of primary *FANCD1* deficient cells to undergo HDR using ODNs as a template.

### 2.3. Phenotypic Rescue of Gene Corrected Primary Cells

**Figure 4:**
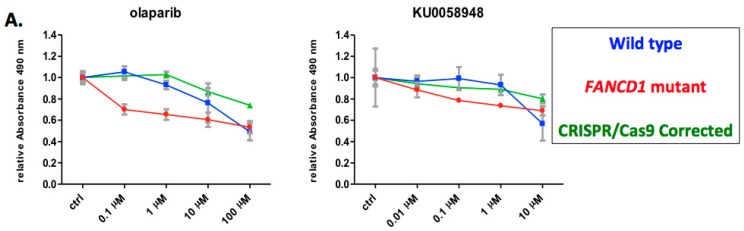

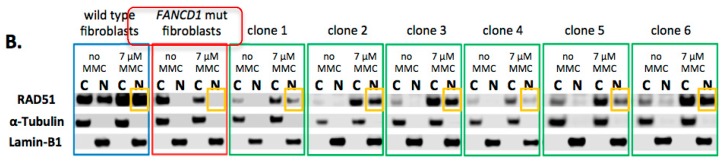
*FANCD1* functional assessment. (**A**) Cellular viability assay. Triplicate samples of gene corrected clones were assessed for viability in response to the PARP inhibitors olaparib or KU0058948. Tetrazolium dye reduction was assessed at 490 nm over four concentrations. Controls were wild type or FANCD1 null fibroblasts of equivalent passage number; (**B**) RAD51 nuclear translocation. Cytoplasmic (“C”) or nuclear (“N”) fractions of untreated or MMC treated cells were analyzed by Western blot for RAD51 localization. Loading controls were α-tubulin or lamin-b1 [28] for the cytoplasmic or nuclear fractions, respectively. Blots are representative of at least two experiments on each of the indicated clones.

We next analyzed the viability of the corrected and uncorrected cell populations by performing an MTS assay over multiple concentrations of olaparib or KU0058948. These data showed that the gene corrected cells maintained long-term viability comparable to wild type cells while uncorrected, parental fibroblasts showed toxicity upon exposure to PARPi (Figure 4A). Previous reports show that functional *FANCD1* is required for nuclear localization of RAD51 [26,27]; therefore we assessed the subcellular localization of RAD51 following mitomycin-C (MMC) induced DNA damage. Western blot analysis of cytosolic and nuclear fractions showed a failure of RAD51 nuclear localization in *FANCD1* null parental cells (Figure 4B.) One corrected clone was unavilable for further analysis due to spontaneous senescence; however, the remaining six CRISPR/Cas9 corrected clones showed retsored nuclear translocation of RAD51 (Figure 4B). These data show that HDR gene correction also resulted in functional rescue of the FA phenotype. 

### 2.4. CRISPR/Cas9 off Target Analysis

The promiscuous activity of programmable nucleases at sites of overlapping sequence homology is an important consideration for their safe application. To assess this we performed an in silico screen to identify putative sites within coding regions of the human genome (Table 1). 

**Figure 5:**
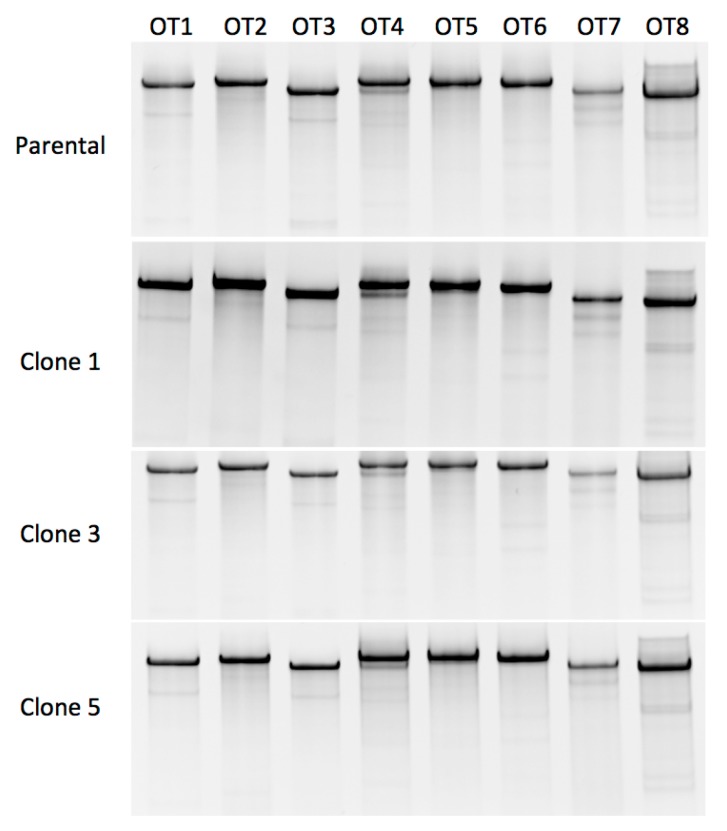
*FANCD1* CRISPR/Cas9 reagent off target analysis. At top is the Surveyor analysis for the primary parental fibroblasts. Three gene corrected clones (1, 3, and 5) were also assessed. OT 1–8 corresponds to the genes listed in Table 1.

To determine whether the *FANCD1* reagent exhibited off target activity we performed Surveyor analysis for the eight predicted intragenic sites in FA cells (uncorrected parental line and three corrected clones). No apparent off target activity was observed at the resolution (~1%) of the Surveyor methodology (Figure 5). To further analyze whether off target events occurred, we utilized 293T cells operating under the hypothesis that the chromosomal rearrangements and aneuploidy observed in this population may present greater opportunity for off target nuclease effects to manifest. Similar to the primary cell data, we did not observe promiscuous nuclease activity (Figure S4). These data show that the safety profile of the designed reagent is favorable with an apparent absence of off target double stranded break induction in the analyzed sites.

## 3. Discussion

We report here the first demonstration of gene editing of FA primary cells and the selection of a homogenous population of gene edited clones using PARP inhibitors. To date, the *FANCA*, *FANCC*, and *FANCI* genes have been targeted for correction with ZFNs, TALENs, or CRISPR/Cas9 [14,15,16]. A requirement for successful gene editing in each of these studies was a requirement for exogenous expression of telomerase reverse transcriptase (*TERT*) or pluripotency inducing reprogramming factors [14,15,16]. Our previous work for *FANCC* gene correction required lentiviral transduction of patient cells with a human TERT (hTERT) construct [15]. This allowed for clonal derivation of CRISPR/Cas9 modified cells using a donor that contained a puromycin selection marker. The combination of h*TERT* and puromycin facilitated obtainment of homogenous clones without senescence while parallel studies in *FANCC* cells lacking h*TERT* showed high levels of senescence. These previous studies required subsequent removal of the puromycin cassette representing a second gene transfer and clonal isolation/screening step. As an elegant methodology to maximize the benefits of h*TERT* expression, Rio and colleagues utilized cre recombinase excisable cassettes in *FANCA* cells such that transient *TERT* could be employed to achieve gene editing with subsequent removal [14]. Further, their floxed reprogramming gene construct could also be selectively removed. However, similar to our necessity of further modification to remove puromycin their strategy also required cre recombinase addition in order to excise the foreign transgene [14]. To extend these foundational studies we undertook efforts in *FANCI* deficient cells to introduce non-integrating reprogramming factors and a donor reliant on gene correction and application of the phenotypic selective marker MMC [16]. Similar to *FANCA* and *FANCC* fibroblasts the primary *FANCI* fibroblasts were recalcitrant to gene correction and clonal derivation [16]. In contrast, pluripotent cells obtained using Sendai viral reprogramming were amenable to gene correction and selection with MMC [16]. The use of MMC; however, impaired the stem cell potential of the cells mandating our use of a floxed puromycin selection marker [16]. Collectively these studies show the ability of FA cells to undergo gene targeting using programmable reagents; however, a persistent hurdle has been primary cell correction with a selection strategy using a clinically viable reagent. 

To address the lack of correction of FA primary cells in the literature we implemented a line of study using *FANCD1* deficient cells. The choice of FA pathway compromisation was carefully considered and *FANCD1* is a severe form of FA as well as is a common gene that is mutated in multiple tumors [29]. The clinical impact of this gene is significant and we developed a CRISPR/Cas9 reagent to target a dinucleotide deletion in exon 8 (Figure 1). We observed robust activity and the proximity of the CRISPR/Cas9 site to the mutation allowed us to employ an oligonucleotide (ODN) donor-based strategy. Sense or anti-sense versions of the ODN donor were tested and showed that the sense configuration mediated higher gene correction rates than the anti-sense counterpart (Figure 3A and Figure S2). This strand preference is in keeping with previous reports [30,31] and the ability to achieve HDR from an ODN template is highly significant. In the context of FA, integrase deficient virus, adenoassociated virus, or plasmid DNA have been employed for gene correction [14,15,16]. Viral based vectors require production using packaging cell lines and plasmids that can be laborious, non-uniform in regards to titer/amount produced, and are associated with significant costs for clinical scale up and application. Likewise, plasmid DNA requires specialized production for translational application and random integrants can result in genomic disruption. In contrast, the GMP production of ODNs is favorable both in cost and scale and the addition of phosphorothioate modifications can increase ODN stability such that more ODN template persists for serving as an HDR template. In our donor design strategy, we incorporated silent mutations to prevent nuclease re-cutting of the modified locus, the GT dinucleotide that is deleted in the *FANCD1* parental population, and introduced a restriction enzyme site to facilitate better detection of gene modified loci (Figure S3). Under these conditions we observed HDR (Figure 2 and Figure 3) in a polyclonal population of cells that underwent selection. The selective pressure consisted of puromycin, olaparib, or KU0058948. Because the pX459 plasmid contains a puromycin resistance gene we transiently selected with puromycin to enrich for cells that underwent successful gene transfer. Selection by this method has been shown to promote modified cell outgrowth [29]; however, we did not see increased rates of HDR following this transient treatment (Figure 3A). Rather, sequencing of the *FANCD1* locus revealed insertions/deletions consistent with non-homologous endjoining-based repair (data not shown). Olaparib is a widely employed PARP inhibitor and is employed for tumor therapy due to its selective lethality in BRCA defective cells [32]. Normally PARP-1 is activated by DNA breaks resulting in progressive addition of ADP-ribose polymeric scaffolds that recruit mediators of DNA break repair [33]. We hypothesized that olaparib or use of its analog KU58948 would selectively deplete uncorrected, BRCA deficient *FANCD1* cells and enrich for HDR corrected events. Indeed, we were able to select and derive seven clones, only one of which underwent senescence (Figure 3). The remaining six clones showed the ability to proliferate in the presence of DNA damaging agents (Figure 4A). In contrast, previous work using MMC as a selection agent showed significant senescence and concomitant impairment of cellular function [15,16]. Toward determining whether the derivative clones were phenotypically restored we performed Western blot analysis on cytosolic and nuclear fractions for RAD51 following DNA damage induction with MMC. RAD51 plays a key role in DNA break repair and its nuclear localization is dependent on FANCD1 [34]. In uncorrected, parental cells we observed cytoplasmic sequestration in the absence or presence of MMC (Figure 4B). Each of the six corrected clones showed nuclear translocation of RAD51 (Figure 4B). Taken together these data show the usefulness of PARP inhibition as a clinically viable selection agent for the recovery of gene corrected cells that are phenotypically rescued. Follow on studies will address the effect PARPi has on the cellular phenotype in translational engineering in HSPC and induced pluripotent stem cells (iPSC). The effect of PARPi on HSPC in vivo is of great interest particularly in light of recent studies [35] showing that malignant transformation of HSPC results in greater rates of DNA damage and potential sensitivity to PARPi. This suggests a possibility of genome modified HSPCs surviving and expanding in the presence of PARPi that will also confer added benefit by depleting transformed HSPC and their progeny.

An important consideration, particularly in DNA damage repair defective disorders, is the specificity of DNA break induction mediated by a nuclease. To assess this, we rank ordered the potential off target sites using an in silico predictive algorithm [34,36]. Using the Surveyor method, we assessed whether promiscuous nuclease activity was prevalent. None of the eight predicted off target sites showed evidence of ectopic Cas9 activity strongly suggesting a highly specific reagent (Table 1, Figure 5 and Figure S4).

Because bone marrow failure is a life-threatening complication of FA the current standard of care is allogeneic hematopoietic cell transplant. Despite advances in improving outcomes for allogeneic recipients in regards to the source of the graft and the conditioning regimen, severe side effects can still occur [37,38,39]. The ultimate goal is to engineer hematopoietic stem and progenitor cells (HSPC) for autologous ex vivo therapy. Current lentiviral gene therapy phase I clinical trials show that the intervention is well tolerated in regards to safety (*n* = 2 patients in NCT01331018); however, the number of transduced cells rapidly diminishes in the periphery [40,41]. Moreover, the potential for vector insertional mutagenesis [42,43], clonal dominance [44], and unregulated expression of a DNA repair protein with the potential for apoptotic resistance [45] in the FA pre-malignant phenotype makes gene editing highly desirable. We were able to effectively modify primary cells as part of an autologous strategy. Because of the limiting numbers of HSPC in FA patients the use of them for pre-clinical optimization is limiting. Therefore, the choice of cell population for reagent development, optimization, and deployment is crucial. We have employed both fibroblasts and iPSC for FA gene editing modeling. Due to their increased cell division rates, iPSC can undergo high rates of HDR; however many FA complementation group defects show a poor ability to undergo reprogramming in the absence of a functional FA repair pathway [46]. *FANCD1* cells were not analyzed in this previous study [46] but given the dramatically lower rates of reprogramming efficiency in FA compromised cells the ability to correct primary fibroblasts is highly significant. Their low replicative capacity, gene transfer and HDR rates more closely mimic HSPC than do iPSC making them a relevant platform for modeling gene editing in support of extension to HSPC. Further, new avenues in cellular engineering show the potential of iPSC to serve as a platform for hematopoietic progenitor development [47] making fibroblasts a decidedly relevant tool of discovery for downstream regenerative approaches.

In sum, nuclease-based modification of the genome in FA along with selection promotes obtainment of corrected cells. Importantly, cells treated with PARPi alone did not result in spontaneous correction of the FA gene. Revertant mosaicism has been observed in FA [48,49,50,51] raising the possibility of applying selective pressure without need of nucleases for modification. However, the time in which it takes for spontaneous correction to occur may be offset by the corresponding accumulation of mutations at other loci. In contrast, nucleases promote dramatically higher HDR rates compared to their absence [52,53] and accomplish gene repair in a highly specific manner. We propose that this represents an ideal strategy for the accelerated generation of normalized cells such that genomic insults that are manifest or acquired via the cell derivation, modification, and expansion process are minimized. A corresponding consideration is the ability to construct an allele specific reagent that would allow for use of the normal portion of the opposite allele in the context of a compound heterozygote as part of a “natural” donor template. In our current study the mutation site does not allow for preferential recognition by CRISPR/Cas9 because the nuclease target site is outside of the mutation site (Figure 1). Future studies will assess the ability of CRISPR/Cas9 to target mutated alleles and can employ PAM variant Cas9 candidates [54,55] in order to overlap the mutation and nuclease sequences in order to confer allele specificity. Such an approach is intriguing as it may obviate the need for an exogenous donor.

## 4. Materials and Methods

### 4.1. Patient Samples

The Declaration of Helsinki requirements for research on human subjects was followed and approval of the University of Minnesota Institutional Review Board Human Subjects Committee was granted (authorization designator: 1301M26601). Following this, and with informed consent, a skin punch biopsy from a patient with FANCD1 gene mutations was obtained (Figure 1A). Fibroblasts from human healthy dermal neonatal fibroblasts and embryonic kidney 293T cell line were purchased from American Type Culture Collection (ATCC; Manassas, VA, USA).

### 4.2. Culture Conditions

293T cells were cultured in Dulbecco’s Modified Eagle‘s Medium with 10% fetal bovine serum (FBS), Glutamax (2 mM) and penicillin/streptomycin (0.1 mg/mL each). Fibroblasts were cultured in Minimum Essential Medium Eagle (Millipore-Sigma, St. Louis, MO, USA) with 20% FBS, nonessential amino acids (100 U/mL), Glutamax (2 mM), penicillin/streptomycin (0.1 mg/mL each), epidermal growth factor and fibroblast growth factor (10 ng/mL each), and antioxidant supplement (all Thermo Fisher Scientific, Waltham, MA, USA or Millipore-Sigma, St. Louis, MO, USA). Cells were maintained at 37 °C and 5% CO_2_; FA fibroblasts at low oxygen conditions (2% O_2_).

### 4.3. CRISPR/Cas9 Design

A guide RNA of the CRISPR/Cas9 nuclease was synthesized as a ssDNA oligonucleotide and cloned into pSpCas9(BB)-2A-Puro (PX459) V2.0 (a gift from Feng Zhang; Addgene plasmid # 62988) by Gibson assembly [56]. The resulting CRISPR/Cas9 plasmid was transfected into 293T cells by Lipofectamine 3000 (Thermo Fisher Scientific, Waltham, MA, USA) and harvested 72 h later. DNA from 293T cells was PCR-amplified with primers covering CRISPR/Cas9 cut site: followed by Surveyor nuclease assay (IDT, Coralville, IA, USA) performed per the manufacturer′s guidelines. 

### 4.4. Gene Editing in Primary Cells

CRISPR-Cas9 plasmid (1 µg) and corrective donors synthesized as ssDNA oligonucleotides (10 ng each; IDT) were electroporated into fibroblasts by Neon transfection system (1500 V, 1 pulse, 20 ms width; Thermo Fisher Scientific, Waltham, MA, USA).

### 4.5. Selection

Selection of the gene-edited population was done either 24 h post electroporation by puromycin (for 72 h; 0.4, 0.7 and 1.1 µM; EMD Millipore, Billerica, MA, USA) or 72 h post electroporation by PARPi (for 7 days; olaparib (0.1, 1 µM; ApexBio, Houston, TX, USA), KU0058948 (0.1, 1 µM; Axon Medchem, Reston, VA, USA)). Monoclonal populations were prepared by plating the gene-edited cells at low density (300 cells per 10-cm dish). Forty-eight hours later, glass-cloning cylinders were placed around single-cell colonies using sterile silicon grease. Clonal populations were progressively transferred into larger wells/flasks. Selected cells were analyzed by PCR. For HDR donor specific detection: F: 5′-TGAAGAAGCTTCCGAAACCG-3′ and R: 5′-GCCACACAGTGCACCATAGA-3′. For locus amplification and subsequent donor detection by *Hind*III digest the following primers were used: PCR F: 5′-CACCAAGCCATATCTTACCACC-3′, PCR R: 5′-ACAGCAGAGTTTCACAGGAAGT-3′. PCR was performed at: 95 °C × 2 min, 40 cycles of 95 °C × 45 s, 57 °C × 45 s, and 68 °C × 1 min.

### 4.6. Off-Target Analysis

CRISPR/Cas9 of target cutting sites were identified using the MIT CRISPR Design tool and the CRISPOR tool [57]. Lipofectamine-based gene transfer of the FANCD1 gRNA and Cas9 in FANCD1 corrected clones and 293 cells were utilized for analysis for on and off target activity using the primers below and amplification with AccuPrime DNA polymerase (ThermoFisher) at: 95 °C × 2 min and 35 cycles of 95 °C × 40 min, 58.5 °C × 40 s, 68 °C × 1 min. Surveyor assay was then performed using amplicons generated with the below primers (shown 5′–3′) followed by PAGE electrophoresis.

*FANCD1* surveyor FAAACTTTATCACAGGGTATGTGCTT*FANCD1* surveyor RCAGCATCATCTGACTTTCCAA*FANCD1* OT 1 Surveyor FCCAGACCAGAAACCGAAAAA*FANCD1* OT 1 Surveyor RTGGCAGTTTGTCCATTTGAA*FANCD1* OT 2 Surveyor FCATCCTGAAAAATGATGGGATT*FANCD1* OT 2 Surveyor RATCTTCCTCCCTTCCTCCTG*FANCD1* OT 3 Surveyor FCCCCCAACTACATTCGAAAA*FANCD1* OT 3 Surveyor RAATTTGGTGGGTTCTACTTGTTT*FANCD1* OT 4 Surveyor FTGAACGTCAGAAGGGCTAGAA*FANCD1* OT 4 Surveyor RGACGTCAAGGTTGCAGTGAA*FANCD1* OT 5 Surveyor FGGCCAGTGGTTCTCAACTTT*FANCD1* OT 5 Surveyor RTGTTCCCATGAGTTTTGTGG*FANCD1* OT 6 Surveyor FACAAACTGCCGAACAAGAGG*FANCD1* OT 6 Surveyor RAGGCTGAGTGGTACTCCATTG*FANCD1* OT 7 Surveyor FTTGTGAATGAGGTGAGATGAGG*FANCD1* OT 7 Surveyor RGATCTTGGCTCACTGCAACC*FANCD1* OT 8 Surveyor FCATATTGTCTGGGTGCCACA*FANCD1* OT 8 Surveyor RTCACCACAACCCCATAAAGC

### 4.7. FANCD1 Functional Assay

Nuclear and cytoplasmic protein fractions from cells cultured with Mitomycin C (7 µM for 1 h followed by 18 h of recovery) were separated by gel electrophoresis, transferred to Nitrocellulose membrane and incubated overnight with the anti-FANCD1 antibody (51RAD01 (3C10), 1:200, Thermo-Fisher Scientific, Waltham, MA, USA). The purity of protein fractions was confirmed by staining with anti α-Tubulin (TU-01, 1:1000, Exbio, Vestec, Czech Republic) and anti Lamin-B1 (sc-6216, 1:200, Santa Cruz Biotechnology, Dallas, TX, USA) antibodies.

### 4.8. Traffic Light Reporter Assay

Traffic light reporter assay was performed as previously described [15,24] following delivery of equimolar amounts of an ODN or dsDNA GFP repair template and CRISPR/Cas9 nuclease.

### 4.9. Positive and Negative Strand Oligonucleotide Donor Assessment

Cas9 nuclease/gRNA plasmid and ssODNs for sense or anti-sense (sequence below) were delivered to 293T cells by lipofection. At 48 h a three primer PCR was performed using the locus specific primers: F: 5′-CACCAAGCCATATCTTACCACC-3′, PCR R: 5′-ACAGCAGAGTTTCACAGGAAGT-3′ and a donor specific primer: 5′-GGATCCAAGCTTCGTCGACCTAGCC-3′ 

Sense donor. 5′-TTGCATTCTAGTGATAATATACAATACACATAAATTTTTATCTTACAGTCAGAAATGAAGAAGCATCTGAAACTGTATTTGTACGGATCCAAGCTTCGTCGACCTAGCCTAAATATGACATTGATTAGACTGTTGAAATTGCTAACAATTTTGGAATGCCTTGTTAAATTATTTATCTTACATTTTTAA-3′ 

Anti-sense donor. 5′-TTAAAAATGTAAGATAAATAATTTAACAAGGCATTCCAAAATTGTTAGCAATTTCAACAGTCTAATCAATGTCATATTTAGGCTAGGTCGACGAAGCTTGGATCCGTACAAATACAGTTTCAGATGCTTCTTCATTTCTGACTGTAAGATAAAAATTTATGTGTATTGTATATTATCACTAGAATGCAA-3′. Bold underlined sequences show the portion recognized by the donor primer.

### 4.10. Graphics

Illustrations were generated using templates from Motifolio Inc. (Ellicott City, MD, USA).

## 5. Conclusions

We demonstrate primary Fanconi anemia cell gene correction by gene editing for the first time. The experimental parameters described herein are highly relevant to translational FA cellular engineering and are additive to the fields of FA and gene editing.

## Figures and Tables

**Table 1:** Off target sites present in coding regions.

Off Target Candidate	5′-Target Sequence-3′	Gene	Mismatches
	CTTACAGCAGTAGTATCATGAGG	*FANCD1*	X
1	TTTGCAGGAGCAGTATCATGAAG	*GATA3*	4
2	CTTACAGTACTAGCATCATGGGG	*AFF2*	3
3	CTTACTGAAGTAGTCTCAGGAAG	*PATL1*	5
4	CTTACAGAAGTATTATCACCGGG	*MFAP1*	5
5	TTTACAGCAGCAGTAGAATGGAG	*NUMB*	6
6	CTTACTGCAGAAGTTTCATCCAG	*TCERG1*	6
7	CGCACAGCAGTAGCATCCTGGAG	*USP54*	6
8	CTTGCAGCAGGAGGATCGTGCAG	*SPON1*	6

The target sites are shown at left with mismatches between the on and off target sites shown in red. Center panel indicates the gene the off target site is located and the total number of mismatches between *FANCD1* and the off target sites are shown at right.

## Supplementary Materials

Supplementary materials can be found at www.mdpi.com/1422-0067/18/6/1269/s1.

## Abbreviations

FA	Fanconi anemia
CRISPR/Cas9	clustered regularly interspaced short palindromic repeats/Cas9
BMF	bone marrow failure
ZFN	zinc-finger nucleases
TALEN	transcription activator-like effector nucleases
PARP	poly(ADP-ribose) polymerase
PARPi	PARP inhibition
gRNA	guide RNA
NHEJ	non-homologous endjoining
ODN	oligonucleotide donor
SNP	single-nucleotide polymorphisms
HDR	Homology directed repair
MMC	mitomycin-C
TERT	telomerase reverse transcriptase
HSPC	hematopoietic stem and progenitor cells
iPSC	Induced pluripotent stem cells
