# Supplementary material for: CRISPR/Cas9-Mediated Correction of the FANCD1 Gene in Primary Patient Cells
Source: Int J Mol Sci. 2017 Jun 14;18(6):1269. doi: 10.3390/ijms18061269 (PMC5486091; doi:10.3390/ijms18061269)
Supplement: Supplementary file 1 [file ijms-18-01269-s001.pdf]

CRISPR/Cas9-Mediated Correction of the *FANCD1* Gene in Primary Patient Cells.

Karolina Skvarova Kramarzova [1,2], Mark J. Osborn [1,3,4,5,*], Beau R. Webber [1], Anthony P. DeFeo [1], Amber N. McElroy [1], Chong Jai Kim [6] and Jakub Tolar [1,3,5]

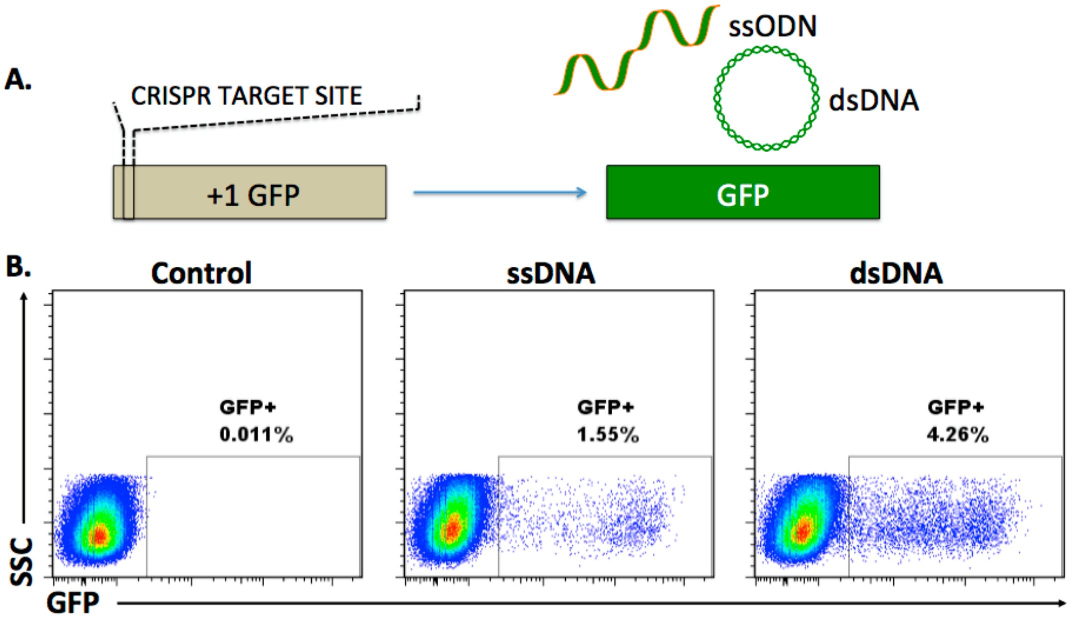

Figure S1. Traffic light reporter (TLR) assay. (**A**) A previously described single copy integrant [57] containing a nuclease target site was utilized. The TLR construct has in interrupted GFP that is out of frame by one base and therefore inactive (left). HDR restores GFP expression and ss or dsDNA donors were employed; (**B**) 293T cells that were untreated (left), or received an ssODN (middle) or dsDNA donor (right) were measure for HDR restored GFP expression by flow cytometry. Side scatter is shown on the *y*-axis and GFP is on *x*-axis. Percent GFP is shown above the FACS gate.

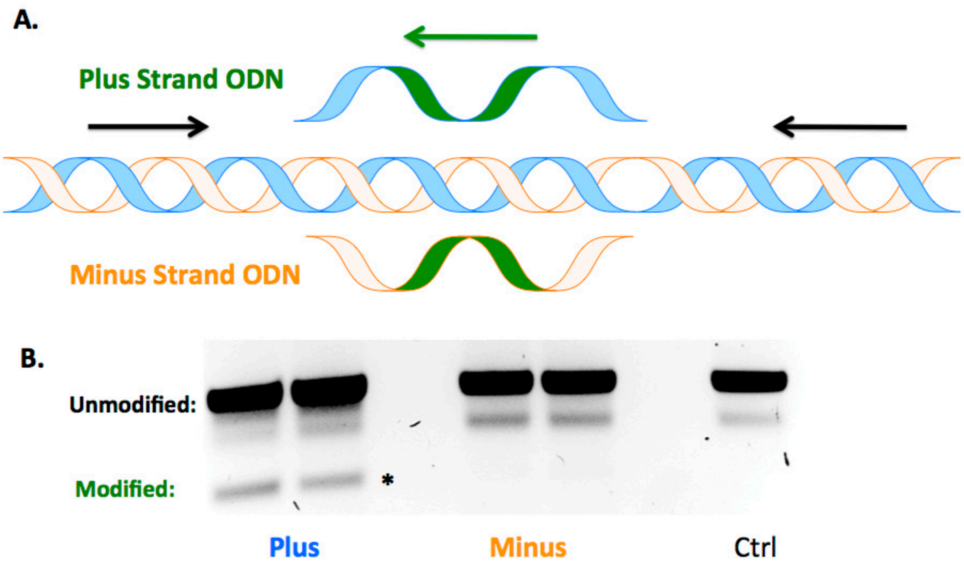

Figure S2. Strand preference for DNA repair. (**A**) A plus or minus strand donor were introduced into 293T cells with the Cas9 nuclease and guide RNA. The ODN contains a unique sequence not present

in the human genome (green portion of donor). At 48 h a three primer PCR was performed using locus specific primers outside the donor (black arrows) and a donor specific primer (green arrow); (**B**) Unmodified alleles are shown at top and are the result of amplification with the locus specific primers. HDR results in a smaller PCR product with amplification between the green and black arrow (identified with asterisk). Duplicate samples are shown with a prominent band observed in cells treated with a donor targeting the plus strand.

sense ssDNA oligodonor (5'→3')

c*a*atacacataaattttatcttacagTCAGAAATGAAGAAGCTTCCGAAACCGTATTCCCTCACGAC
ACTACTGCTgtaagtaaatatgacattgattagactgttgaaattgctaac*a*a

antisense ssDNA oligodonor (5'→3')

t*t*gttagcaatttcaacagtctaatcaatgtcatatttacttacAGCAGTAGTGTCGTGAGGGAATA
CGGTTTCGGAAGCTTCTTCATTTCTGActgtaagataaaatttatgtgtat*t*g

Figure S3. *FANCD1* oligonucleotide donors. Sequence of sense and antisense ssDNA oligonucleotide donors are shown. Brown box depicts exon 8 of the *FANCD1* gene, blue box represents restored dinucleotide GT. Red nucleotides are silent SNPs introduced into the donor sequence. Asterisks are the phosphorothioate modifications.

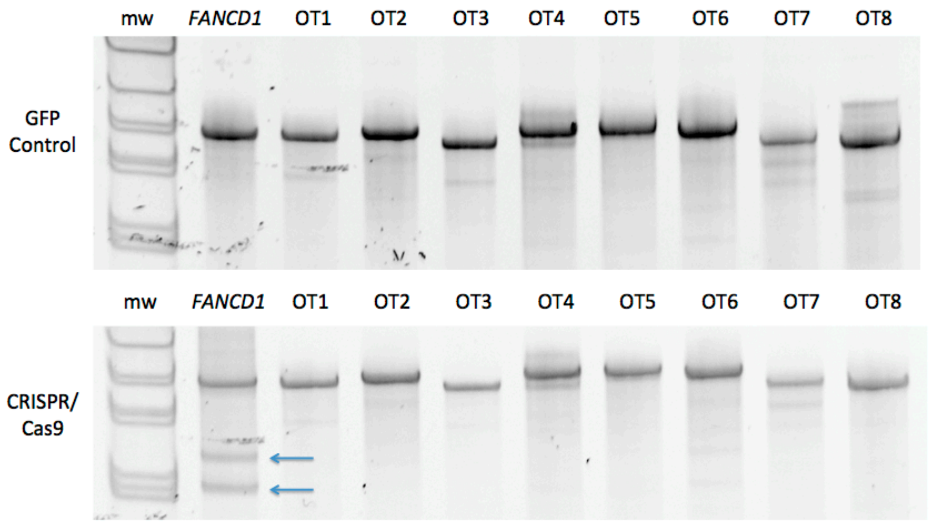

Figure S4. *FANCD1* nuclease off target analysis in 293T cells. At top is the Surveyor analysis for 293T cells treated with a GFP plasmid plus the *FANCD1* gRNA. At bottom are *FANCD1* gRNA plus CRISPR/Cas9 treated 293T cells. OT 1-8 corresponds to the genes listed in Table 1. Blue arrows show fragmentation products consistent with nuclease activity at the *FANCD1* locus.
